# A Regulatory Risk-Based Approach to ATMP/CGT Development: Integrating Scientific Challenges With Current Regulatory Expectations

**DOI:** 10.3389/fmed.2022.855100

**Published:** 2022-05-13

**Authors:** Laura I. Salazar-Fontana

**Affiliations:** ^1^LAIZ Regulatory Science Consulting, Lausanne, Switzerland; ^2^Immunogenicity Integrated, Nay, France; ^3^NDA Group Advisory Board, Surrey, United Kingdom

**Keywords:** advanced therapy medicinal products, cell and gene therapy products, gene therapy, cell therapy, FDA, EMA, regulatory risk, regulatory science

## Abstract

Cell and Gene Therapy Products (CGT), regulated as Advanced Therapy Medicinal Products (ATMP) in the European Union (EU), represent a novel and varied group of biotherapeutics developed to treat specific conditions for which there are limited or no effective treatments. The novelty and complexity of this product modality demands a regulatory risk-based approach to define a sound development plan, particularly, as most developers aim to target more than one market area simultaneously for clinical development and registration. This regulatory strategy should be built on solid scientific data that also addresses general regulatory recommendations to enable a benefit:risk analysis that is aligned with the particularities of each CGT product. This risk-based approach is especially helpful when no detailed product-specific regulatory guidelines are available. The goal of this article is to orient developers on how to build a combined EU/US regulatory strategy through the assessment of commonly understood quality (CMC), non-clinical, and clinical regulatory risks faced by ATMP/CGT.

## European Union and United States Regulatory Landscape for ATMP/CGT Products

In the United States (US), the Food and Drug Administration regulates CGT as “biological products” as defined in section 351(i) of the Public Health Service Act (PHS), 42 U.S.C. 262 (i) (1); therefore, both genetically modified cells and gene therapies are evaluated following the same regulatory principles and requirements for quality, safety, and efficacy as biologics. Homologous and minimally manipulated human cells used for implantation, transplantation, infusion, or transfer into a human recipient (Human Cell, Tissues and Cellular and Tissue-Based Products or HCTs/P) follow the “tissue rules” (2). Specific Guidance to industry on FDA’s interpretation of minimal manipulation and homologous use can be found in the 2020 Guidance for Industry HCT- and CTB products (3). This is different in the EU, where Cell and Gene Therapy Medicinal Products, CTMP and GTMP, respectively, and Tissue Engineered Products (TEP), are regulated as Advanced Therapy Medicinal Products (ATMP) (4). The definition of a TEP is given in the ATMP regulation (5), whereas the definitions of the CTMP and GTMP, are described in the Directive 2009/120/EC (4). Please note that the classification of TEP is based on the proposed mechanism of action (MoA) rather than on the level of manipulation of the primary cell population or tissue used in its manufacture.

Despite specific regulatory nuances, both the US and the EU regulatory authorities, the FDA and the European Medicines Agency (EMA) have recognized the uniqueness and complexity of ATMP/CGT development and have subsequently published multiple guidelines on specific expectations on the quality (CMC), non-clinical, and clinical considerations of ATMP/CGT (6, 7). Developers are strongly advised to consult these guidelines when identifying and assessing the particular regulatory risks linked to their product.

Newly created regulatory pathways, like the Regenerative Medicine Advanced Therapy (RMAT) designation (8) in the US and Priority Medicines scheme (PRIME) (9) in the EU, in combination with new meeting modalities have been created to significantly expedite the development process and submissions review timelines. These new regulatory pathways, among other advantages, include more frequent interactions between developers and assessors/regulators to favor the exchange of new scientific and technological advances. Increased communication between developers and regulators have proven to be extremely valuable for areas where there is still limited knowledge.

**FIGURE 1:**
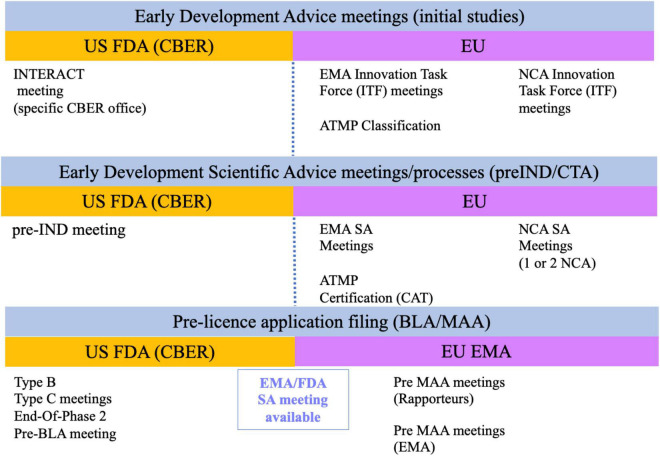
Current formal regulatory meetings with US and EU regulatory agencies.

Both the FDA and the EU National Competent Authorities (NCA) allow requests for informal discussion meetings and scientific advice meetings at minimal or no administrative costs (Figure 1). The Center for Biologics Evaluation and Research (CBER) is the center within the FDA that regulates CGT and HCTs/P. A new meeting modality, called Initial Targeted Engagement for Regulatory Advice on CBER Products (INTERACT) meeting, has been created to promote science-based discussions with the regulators so initial advice can be obtained. Although CBER does not provide official INTERACT meeting minutes, it is highly recommended to incorporate the advice received into the product development plans to reduce regulatory risks when submitting your Investigational New Drug Application (IND) (10). No Prescription Drug User Fee Act (PDUFA) fee is associated with INTERACT meetings. They are granted based on CBER’s availability and resources so they tend to be rarely granted. Note that the INTERACT meeting does not replace the pre-IND and other formal meetings for products regulated under the Prescription Drug User Fee Act (PDUFA) (11). Consequently, it is highly recommended to follow on with a Pre-IND (Type B meeting) meeting prior to submitting your IND application to initiate your First-in-Human (Phase 1) study, especially when seeking guidance on toxicology study designs or completeness of safety monitoring in your Phase 1 clinical study protocol.

A similar pathway to the INTERACT meeting described above exists in the EU. Informal meetings can be requested from each country NCA innovation office or through the centralized Innovation Task Force (ITF) initiative of the EMA. The EMA ITF pathway is also granted based on available resources and may not be available for all applicants (12). Consequently, it may be more advantageous to request scientific advice from one or a couple of NCAs during the early development phase to obtain regulatory feedback on compelling questions (13). A second meeting modality is Scientific Advice (SA) (14). For both the NCA and ITF modalities, the regulatory advice is given through written feedback and may or may not involve a face-to-face meeting with the EMA or NCA authorities depending on the modality. Many developers have usually sought SA from individual NCAs prior to initiating First-In-Human (FIH) studies and later from the EMA centralized procedure when approaching pivotal clinical study(ies), specially when considering an adaptive clinical trial design. This preference may now change thanks to the implementation of the new Clinical Trial Regulation as of beginning of 2022. The new regulation permits the electronic submission of a single Clinical Trial Application (CTA) through the Clinical Trials Information System (CTIS). This means that a single application can be simultaneously assessed by multiple NCA allowing the harmonization of content and assessment (15).

The EMA also offers specific services for small and mid-size enterprises (SMEs) developing ATMPs, such as the classification and the certification of quality and non-clinical data, to help with the identification of gaps that may hamper product authorization. The certification review is conducted by the Committee for Advanced Therapies (CAT) and can be requested at any time of the development, but the highest value is usually obtained before initiating non-clinical or clinical studies (16).

Finally, there is also the possibility to seek joint advice from EMA (17) and FDA (18). This strategy is very useful for advanced programs, such as at the End-Of-Phase2 (EOP2) and/or before finalizing the design of pivotal (Phase 3) clinical studies, to obtain feedback on clinical trial design and selection of clinical endpoints. Please note that the joint nature of the meeting does not imply similar advice from both agencies.

From a regulatory operations perspective, preparing a common template document to approach both EU and US agencies can be extremely helpful, since the supportive documentation for early development advice follow very similar documentation formats. Developers can create a common core document with the relevant overview of the ATMP/CGT program development plan and adapt it according to specific regional regulatory guidelines based on the identified quality, non-clinical and clinical regulatory risks. Clear questions should be arranged based on relevant supportive information so clear regulatory advice can be obtained.

Regarding expediting timelines for product development and the review of the license application, the Orphan Drug Designation (ODD) (EU and US) (19, 20), EU PRIME (9), and US RMAT (8) are of particular interest since they are all aimed to accelerate the development process. For approval of an ODD designation, both in the EU and US, the product needs to meet the criteria for rare condition; this is defined by a prevalence criterion of no more than 5 cases per 10,000 patients in the EU, and approximately 6.25 cases per 10,000 individuals in the US (fewer than 200,000 individuals among the US population), and needs to be substantiated with evidence of non-clinical or clinical efficacy. When the drug does not meet the criteria for ODD but still has the potential to treat a serious condition, developers can apply for other regulatory designations to speed up the regulatory process. In the US, the RMAT designation was issued on December 2016, as part of the 21st Century Cures Act. This designation can be requested at the time of the IND submission or as an amendment to it. An investigational drug is eligible for RMAT designation if it meets the following criteria: the definition of regenerative medicine; intends to treat, modify, reverse, or cure a serious condition, and preliminary clinical evidence indicates its potential to address an unmet medical need. RMAT designation shares the same features as a breakthrough therapy, such as actions to expedite their development and its review and a rolling review process, but it also includes early discussions on potential surrogate or intermediate endpoints, as ways to support accelerated approval (21). In the EU, the PRIME scheme is the close equivalent to the RMAT designation in the US (9). Acceptance of a program under the PRIME scheme requires demonstration of a major therapeutic advantage over existing treatments or benefit to patients without treatment options. An application that is granted PRIME scheme is entitled to the early nomination of a CHMP or CAT rapporteur, SA meetings with additional stakeholders at key milestones and the designation of a dedicated contact point and accelerated assessment of the market application. Breyanzi is an example of the first CAR T therapy that was granted RMAT designation and PRIME scheme (22).

When the criteria for RMAT and/or PRIME are not fulfilled, it is still possible to expedite the Biologics License Application (BLA)/Market Authorization Application (MAA) review process through other accelerated regulatory paths. In the US, four [4] distinct pathways are available to speed up market availability of drugs aimed to treat serious conditions for which there may or not be an available treatment; these are: Priority Review, Breakthrough Therapy, Accelerated approval, and Fast track (21). In the EU, it is also possible to request an accelerated assessment (23) at least 2–3 months prior to submitting the MAA, although it is highly recommended to seek regulatory advice through a pre-submission meeting prior to the MAA submission to ensure completeness of the dossier. Conditional approval is the EU equivalent to the US Fast-Track designation, and allows submission of final prove of efficacy under specific obligations with defined timelines post-MAA filing. It is worth mentioning that although both the accelerated assessment (EU) and priority review (US) processes characterize by the use of shortened review timelines and faster decision making, they demand the same level of quality evidence and scientific/medical standard for approval than a standard MAA and BLA. This requirement can pose significant constraints to developers and should be carefully planned.

For ultrarare conditions with very limited number of patients, it is also possible to apply for MAA under exceptional circumstances. There are strict legal provisions for both conditional MA and MA approval under exceptional circumstances (24). There are several ATMP approved under conditional MA (i.e., Zolgensma, Tecartus) and one, Glybera, approved under exceptional circumstances but later recalled from the EU market (25–27). In the US, a fast-track designation grants sponsors the possibility of a priority review process, which means that FDA will take an action within 6 months of submission instead of 10 months for a standard review. A provision for a rolling submission schedule is also included. This means that the developer can advance the submission of the portions of the BLA application that demonstrate significant improvements in the safety and effectiveness of the new treatment, diagnosis or prevention of a serious conditions compared to standard applications (28). Please note that breakthrough therapies and RMAT designations are eligible for all fast-track designation features including faster review times. Examples of breakthrough and ODD approvals in the US are Kymriah and Yescarta (29, 30).

## De-Risking ATMP/CGT Development

The development of ATMP/CGT products require a considerable amount of scientific innovation. Many of the development strategies applied to their manufacture come with anticipated regulatory risks. For instance, the introduction of novel starting materials such as primary human cells for cell therapies, or the small-scale manufacturing process to meet one batch-one patient supply, or raw materials of research grade, come with challenges that cannot be fully addressed within current regulatory guidelines. This is the scenario where a case-by-case risk assessment evaluation takes central stage. The identification of regulatory risk for each development stage needs to be fine-tuned so suitable evaluation and mitigation strategies can be put in place to address potential regulatory concerns around safety and efficacy.

From a quality (CMC) perspective, one of the major challenges that ATMP/CGT products continue to face is the high degree of variability resulting from both new starting/raw materials and the manufacturing process conditions. So far, all approved autologous Chimeric Antigen Receptor T-cell (CAR T) products are manufactured at a small scale that uses primary cells collected from the patient to be treated. Donor-to-donor variability can be a significant challenge in the standardization of the product quality, especially, when the starting material consists of cells with poor growth kinetics and reduced viability due to the exposure to conventional cancer treatments (i.e., limited numbers of CD8 + T cells for CD19 CAR Ts) (31). Both Novartis and Kite Pharma/Gilead have reported failures in the clinical studies for their autologous CD19-CAR T products Kymriah and Yescarta (32), respectively, due to out-of-specification (OOS) results for critical quality attributes (CQAs) such as the final number of CAR T expressing cells, which, in the worst cases, have left the patient without treatment (31).

The poor viability of the autologous starting material may be better controlled if an allogeneic primary cell source is chosen. Allogeneic cell banks need to be characterized and qualified to a similar extent as the master and the working cell banks (MCB and WCB) used for biologics (33), as defined and the US (34), and for cell sources in the EU (35). Allogeneic cell stocks can also help reduce the donor-to-donor variability. When choosing an allogeneic cell source, it is highly recommended to define donor selection criteria and a good characterization to warrant comparability of cellular characteristics between different donors and control of the cells during manufacturing. A strategy currently being explored for developing “off-the-shelf” allogeneic CAR T therapies is the use of pluripotent/undifferentiated human cells. In this case, optimal HLA-matching characteristics should be included to avoid extensive comparability studies and to minimize the risk of rejection in treated patients (36). From a regulatory perspective, it is highly recommended to discuss the criteria for cell source characterization with regulators, especially for establishing allogeneic cell banks since expectations may vary between FDA and EMA.

Coming back to the manufacturing process, the use of biological starting materials, such as growth factors (serum, growth factors, cytokines), production or packaging cell lines, plasmids and viruses is very common for ATMP/CGT. These materials can introduce additional product quality variability and should be carefully examined in the initial risk analysis so they can also inform pharmacovigilance mitigation activities (37, 38). General requirements for biological raw/ancillary materials for ATMP/CGT production are detailed in the Ph. Eur. general chapter 5.2.12 (39) and USP >1043< (40). For instance, raw materials of research grade are very often used in the manufacturing of the initial clinical batch. It is highly recommended to discuss the adequacy of your raw material qualification protocols, including risk assessment, with regulators before manufacturing your toxicology and initial clinical batch(es) when using non-GMP raw materials. From a documentation perspective, it is possible for developers to utilize the Drug Master File (DMF) system for certain ancillary materials to support your IND and BLA submissions (41). Please note that no such system exists in the EU and all raw materials must be disclosed and data provided as part of CTA and MAA which will add complexity to the preparation of your dossier.

Gene therapy development entails a different scope of product quality risks. Setting up acceptance criteria limits for CQAs to define strength, potency, and safety may be challenging given the complexity of the manufacturing process and the analytical methodology used for in-process and release testing. The multi-plasmid manufacturing process of Adeno-Associated Virus (AAV) gene therapy products often results in high amounts of empty capsids in the final product which might require modifying the expression vector and the process to improve packaging of the genetic material into AAV capsids (42). AAV viral vectors have an increased risk for eliciting unwanted immunogenicity responses in humans (43). This risk can be exacerbated by the presence of empty capsids. It can also impact product potency and efficacy of the selected dose. Defining strength and potency criteria are critical to link the activity of the product to its *in vivo* effect. The use of a matrixed approach that combines multiple methodologies, such as infectivity and biological activity of the expressed gene in a cell-based assay, is now a well-described expectation in both FDA and EMA ATMP/CGT specific CMC guidelines (37, 44). Potency assays for both cell and gene therapies are expected to be validated before pivotal clinical studies so that a correlation between potency and efficacy can be assessed. A failure to provide a well-validated *in vitro* potency assay representative of the biological activity of the product can become an approvability issue in your license application. Yet, it is highly recommended that the selected assay is fit-for-purpose early in product development for multiple reasons. A robust potency assay will ensure proper activity of the product, help with dose selection extrapolation from non-clinical studies into FIH, and importantly support the demonstration of comparability when moving from non-clinical/clinical batch production to commercial scale (35, 37, 44). Both EU and US authorities will accept validated surrogate assays as long as there is a functional assay available for characterization with correlation to the selected assay(s) during early studies. Thus, the selection of your potency assay format is a regulatory risk that should be carefully leveraged promptly in your development program.

Manufacturing changes occurring during development can also add to the described batch-to-batch variability. According to ICH Q5E (45), generation of batch comparability data should include results from in-process control testing (IPCs), extended characterization, and release and stability testing for both pre- and post-change batches. For cell-based products, such comparability programs can be very challenging because of the small batch size and, consequently, the limited number of retained samples. Regulatory expectations around comparability exercises are similar between the FDA and EMA. EU authorities prefer to see a side-by-side comparison of the results with calculated standard deviations (SD) (46), while US FDA is likely to additionally request a justification for the choice of the statistical approach used for the comparability assessment (47). The defined acceptance criteria limits for your comparability protocol should reflect both process and analytical method variability and be justified by clinical batch data. As for any other biological product, significant differences in quality/activity of the batches and/or changes in specifications during development may involve generation of additional non-clinical and/or clinical data, depending on the level of uncertainty, which can be very difficult when aiming to treat a rare disease or a disorder without a representative animal model. For this reason, it is highly recommended to conduct major manufacturing changes before initiating your pivotal study(ies) since it may not be feasible to gain additional clinical evidence to prove comparable safety and efficacy of the new material.

Let’s now evaluate the potential risks associated with non-clinical studies. From a regulatory perspective, the major difference between the EMA and FDA requirements is the compulsory Genetically Modified Organism (GMO) application in the EU (48). The approval of this application is required for all *in vivo* gene therapy medicinal products including genetically modified cell therapy products and oncolytic viruses before initiating any clinical studies. The separate review of the GMO application needs to be carefully leveraged as part of the combined EU/US regulatory strategy to avoid delays in the initiation of FIH studies. This topic is currently under active debate since it can significantly delay the initiation of clinical studies for ATMP within the EU (49).

Besides the EU regulatory requirement for a GMO application for GTMP and CTMP, the scope of the non-clinical studies is no different from any other biological product. These studies are performed to establish the mechanism of action (MoA), to show proof-of-concept (PoC), and generate evidence on product biodistribution and, more importantly, its safety, in an *in vivo* model, prior to start studies in the human population. For cell therapies, whether we are considering a minimally manipulated or a genetically modified human cellular product, xenogeneic responses derived from the inherent immunological differences between species can render the evaluation of *in vivo* activity and safety inadequate. Under these circumstances, it may be more relevant to explore a combination of *in vivo* responses in humanized or genetically immunosuppressed animals in combination with *in vitro* methods that include human cells or human cell lines (i.e., pluripotent stem cells, cord blood cells, or tumor cells) since they are likely to be more relevant to predict the biological activity and/or on target/off tumor safety signals of the product (37, 50, 51). For CAR T products where the molecular structure of the CAR T molecule is known, immunogenicity prediction tools can help identify the prevalence of linear T cell epitopes in the primary sequence and the species origin of the CDR portion of the molecule so appropriate mitigation strategies can be set to monitor treatment-emergent adaptive immune responses. Risks associated with off target recognition can also be explored using predictive tools such as the membrane proteome array assay (MAP) in the same way as it is used to define the specificity and off-target binding of monoclonal antibody therapies (52). The combination of *in vivo* and *in vitro* methodologies to support demonstration of biological activity, dose selection and regimen, and PoC for cell therapies should be carefully justified and discussed with regulators.

In the case of gene therapies, it is extremely relevant to use an animal model that reproduces the genetic defect aimed to be corrected by the product to avoid misinterpretation of toxicology signals. Toxicology studies are usually conducted in healthy animals. But in the case of a gene therapy product, it may be more relevant to select the same animal model used for pharmacological assessments to avoid magnification of toxicology signals since the overexpression of the gene product can complicate the interpretation of findings. Two significant regulatory safety concerns are associated with gene therapies: one is the oncogenic potential of the viral vectors used to deliver the corrected version of the defective gene, and second is the overexpression of the corrected gene in healthy tissues (off target effect). Regulatory agencies require provisions for long-term safety evaluation of insertional mutagenesis and/or oncogenicity events for up to fifteen [15] years post-treatment (53). The extent of testing is highly dependent on the type of gene editing or delivery system (i.e., transposon elements, CRISPR-Cas 9 gene editing system, AAV vector) and the distribution and persistence profile data collected from non-clinical studies. Annual examinations for the first 5 years are expected followed by 10 years of annual queries or for as long as data indicate that there is no longer risk that needs to be followed. There are slightly different expectations between FDA and EMA beyond the initial 5 years of follow-up evaluations that should be carefully considered (53, 54).

Early clinical development for ATMP/CGT also requires special considerations as most of the time traditional pharmacokinetics (PK) and dose-finding studies are not feasible during FIH studies either because it is a rare condition or because the benefit:risk profile is not acceptable for healthy volunteers. Therefore, dose-selection is usually supported by allometric scaling from PK data collected during non-clinical studies. This approach is likely to be accepted by regulators if scientifically justified and discussed with regulatory authorities prior beginning clinical studies (51, 55). Pediatric populations are in many cases the target population for many gene therapies and it is therefore very likely that preliminary studies in adults are requested by the regulatory agencies when feasible for the condition so a favorable preliminary benefit-risk evaluation can be achieved. ODD should also be considered early during development since both agencies will request information about PoC study results obtained in a specific *in vivo* model of the condition to support the application (20). It is of paramount importance to provide supportive preliminary clinical data obtained in patients affected by the condition for a successful approval of an ODD application when no suitable non-clinical animal model of the disease is available. FDA may consider a combination of alternative data that include the pathogenesis of the disease, a clear description of the drug and its MoA, and supporting *in vitro* data in rare situations. In the EU, it is also necessary to provide supportive data for the life-threatening or chronically debilitating nature of the disease (56).

**FIGURE 2:**
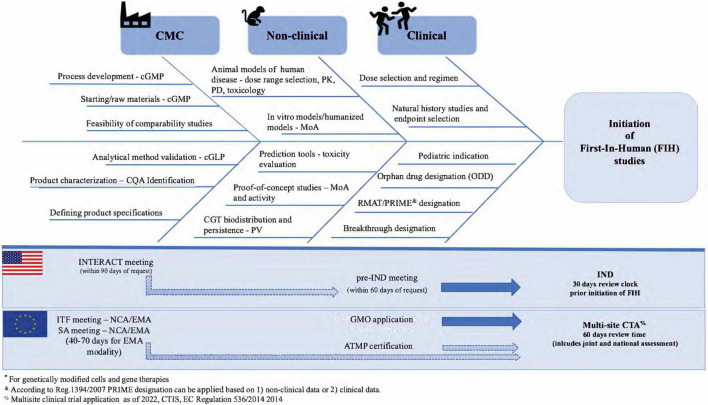
Regulatory risk analysis in early ATMP/CGT development.

In summary, using a risk-based approach to identify quality, non-clinical, and clinical developmental regulatory risks early in ATMP/CGT development that combines current regulatory expectations and regulatory advice opportunities can shorten the time to initiate FIH studies and ultimately obtain licensure (Figure 2). Using this “combined EU/US” regulatory strategy can help to identify regulatory risks, to allocate internal resources and streamline development into two market regions while setting the path for a global submission strategy.

## Conclusion and Discussion

ATMP/CGT belong to today’s fastest growing business segment and scientific innovation areas in life sciences. While de-risking plans may vary between cell and gene therapies, creating an early regulatory strategy that benefits from the existing accelerated regulatory pathways is of paramount relevance to avoid unnecessary delays in clinical development. Developers can benefit from the latest regulatory science practice, scientific advances, and ongoing international coordination efforts amongst agencies to accelerate access to the market without compromising the quality and sound evaluation of safety and efficacy. That is why, preparing a complete regulatory roadmap with defined regulatory milestones for interactions with the EU and US regulatory agencies based on a developmental de-risking approach can maximize efforts and enable access to these personalized medicines to seriously ill patients with no alternative treatments.

## Data Availability Statement

The original contributions presented in the study are included in the article/supplementary material, further inquiries can be directed to the corresponding author.

## Author Contributions

The author confirms being the sole contributor of this work and has approved it for publication.

## Conflict of Interest

LS-F worked as co-founder of Immunogenicity Integrated and NDA Group Advisory Board Member as LAIZ Regulatory Science Consulting Principal.

## Publisher’s Note

All claims expressed in this article are solely those of the authors and do not necessarily represent those of their affiliated organizations, or those of the publisher, the editors and the reviewers. Any product that may be evaluated in this article, or claim that may be made by its manufacturer, is not guaranteed or endorsed by the publisher.
